# Tin Oxide Nanoparticles via Solar Vapor Deposition for Hexavalent Chromium Remediation

**DOI:** 10.1021/acsanm.3c01567

**Published:** 2023-07-07

**Authors:** Konstantinos Simeonidis, Kyriaki Kalaitzidou, Theopoula Asimakidou, Carlos Martinez-Boubeta, Antonios Makridis, Anita Haeussler, Georgios Vourlias, Lluis Balcells

**Affiliations:** † Department of Chemical Engineering, Aristotle University of Thessaloniki, 54124 Thessaloniki, Greece; ‡ Nanotech Solutions S.L., Ctra. Madrid 23, 40150 Villacastin, Spain; § Department of Physics, Aristotle University of Thessaloniki, 54124 Thessaloniki, Greece; ∥ Processes, Materials and Solar Energy Laboratory, CNRS-PROMES, 7 Rue du Four Solaire, 66120 Font-Romeu, France; ⊥ Institut de Ciencia de Materials de Barcelona, CSIC, Campus Universitat Autònoma de Barcelona, A08193 Bellaterra, Spain

**Keywords:** solar-assisted synthesis, drinking water, remediation, nanoparticles, hexavalent chromium

## Abstract

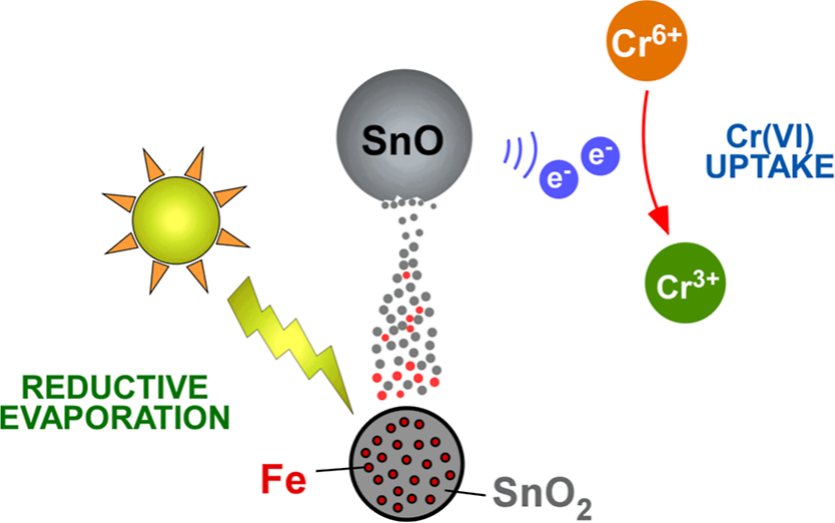

Tin oxide nanoparticles optimized to capture low concentrations of hexavalent chromium from water were developed through a facile, scalable, and low-cost one-step solar vapor deposition methodology. Considering the preservation of high electron donation capacity as the key to support the reduction of mobile Cr(VI) into insoluble forms, the growth of SnO nanoparticles was favored by the co-evaporation of SnO_2_ with Fe powders at various mass ratios. Characterization techniques indicated that the percentage and the stability of SnO is proportional to the Fe content in the target with a requirement of at least 50% wt to inhibit the formation of a passive SnO_2_ surface layer. The produced particles were evaluated regarding their efficiency to capture Cr(VI) under conditions similar to water treatment for drinking purposes (pH 7). It was revealed that passivation-free SnO nanoparticles deliver significant improvement in the adsorption capacity corresponding to the residual concentration of 25 μg/L, reaching a value of 1.74 mg/g for the sample prepared with 50% wt Fe in the target. The increase of water acidity was found responsible for the activation of more reduction sites on the particle surface, as reflected through the elevation of efficiency by more than 20% at pH 6.

## 1 Introduction

The frequent presence of hexavalent chromium Cr(VI) in fresh water resources is deemed one of the major problems of environmental pollution that came to light during the last quarter-century.^1−3^ In the natural reservoirs of drinking water, even small concentrations of Cr(VI) at values of a few dozens of μg/L may be considered a serious threat to the health of long-term consumers.^4^ Such an eventuality has been reinforced by recent findings that correlate exposure to Cr(VI) in drinking water with chronic diseases, cancer, and overall decreased life expectancy.^5,6^ Taking into account these facts, and in response to public pressure, the WHO has issued guidelines for drinking water quality, and a growing number of international policymakers have begun implementing strategies to reduce any relevant risks. Suggestively, California follows a procedure to apply a maximum contaminant level of 10 μg/L by 2024,^7^ whereas European Union member countries agreed to set a limit of 25 μg/L by January 2036.^8^ At the same time, the requirement for communities to identify bodies of water fit for human consumption and take the necessary measures to reduce the level of purification treatment, is expected to intensify the problem of water scarcity in many parts of the world.

The successful treatment of Cr(VI)-contaminated water became a field of intense research effort with the aim of developing and optimizing methods for efficient capture while keeping the respective costs as low as possible.^9,10^ So far, the chemical reduction of Cr(VI) by means of ferrous salts followed by precipitation has been described as the most efficient way to ensure adequate purification of drinking water in large capacity facilities.^11^ However, the need to build large infrastructures with high maintenance, labor, and solid waste handling, discourages further expansion of this technology in medium-scale water treatment units. Consequently, introducing filtration of water through an adsorbent with high affinity to capture Cr(VI) would be an ideal approach to establishing a facile purification method with a high degree of automation. Due to the failure of common water adsorbents (activated carbons and metal oxides) which provide large specific surface areas to operate efficiently in the μg/L range of concentrations, research has focused on the development of materials with electron-donating ability. Such capacity will offer a mechanism for Cr(VI) reduction into insoluble Cr(III) species.^12,13^ Zero-valent iron (ZVI), Fe_3_O_4_, and stannous oxyhydroxides have been reported as the most representative examples of this material category.^14−16^

In this regard, bivalent tin phases contribute two electrons per atom for redox reactions, until they are passivated in the form of Sn^4+^ oxides.^17^ Quite often, a catalysis-boosting effect occurs when the grain size of the material is reduced to the nanoscale, which implies a drastic increase in the specific surface area and the number of active sites per mass of the adsorbent.^18−20^ To this end, the aqueous precipitation route has been reported as a convenient way to synthesize nanosized Sn^2+^ oxyhydroxides. This is despite the fact that the method under discussion suffers from unavoidable aggregation and, in turn, low surface utilization.^21^ To overcome such shortcomings, vapor condensation methods based on the evaporation of target materials at low pressure are preferably applied to obtain well-defined and isolated nanoparticles.^18^ For instance, the solar-powered physical vapor deposition (SPVD) process is a renewable and cost-efficient methodology for the synthesis of nanoparticles with morphologies defined by factors such as pressure, heat of vaporization of the studied material, and solar concentration power.^22^

**Scheme 1:**
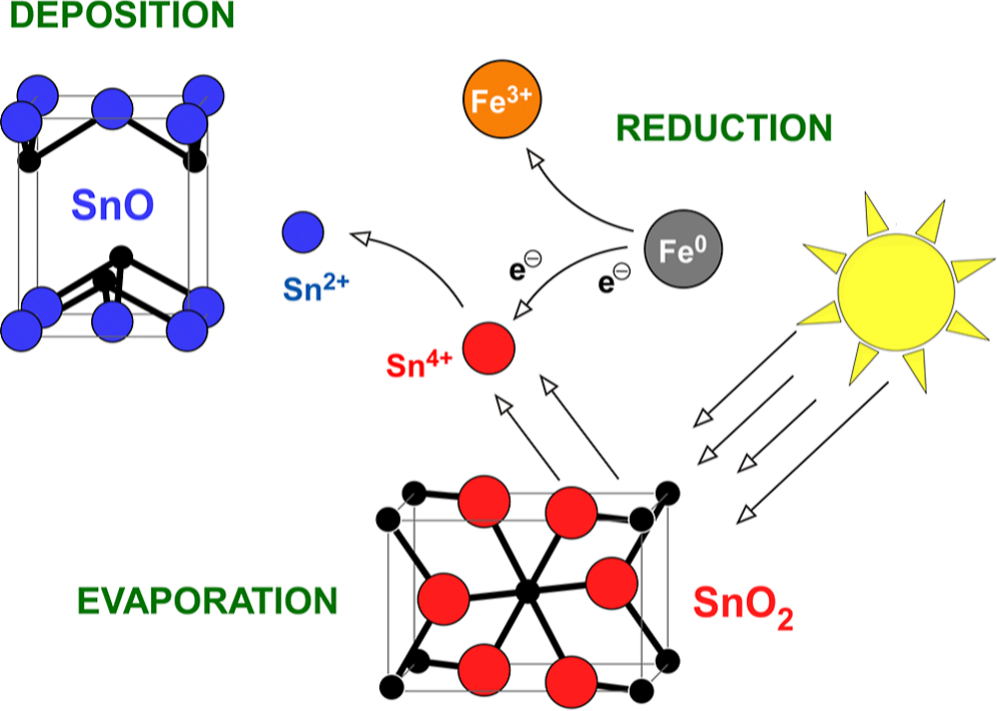
Iron-Assisted Reductive Evaporation of SnO_2_ Toward Production of SnO Nanoparticles

The aim of this study was to develop tin oxide nanoparticles with minimal aggregation, taking advantage of an optimized SPVD configuration to preserve a high reducing potential. According to this concept, chemically stabilized nanoparticles consisting of SnO would become available directly in powder form by transforming cheap raw materials into added-value nanomaterials under a scheme of zero energy consumption. For this purpose, the evaporation of Sn^4+^ oxide powder was combined, for the first time, with the catalytic activity of ZVI to introduce an additional reduction step during the condensation of tin vapors. In this way, nanoparticles composed of high purity Sn^2+^ oxides could be acquired (Scheme 1). These nanomaterials were assessed as potential Cr(VI) adsorbents for the purification of polluted natural-like water, with competitive performance in comparison to previously reported materials. Overall, herein, it is demonstrated that controlling the surface chemistry and the morphology of the SnO_*x*_ nanoparticles is a suitable way to tune their adsorption efficiency.

## 2 Experimental Section

### 2.1 Preparation of Nanoparticles

**Figure 1:**
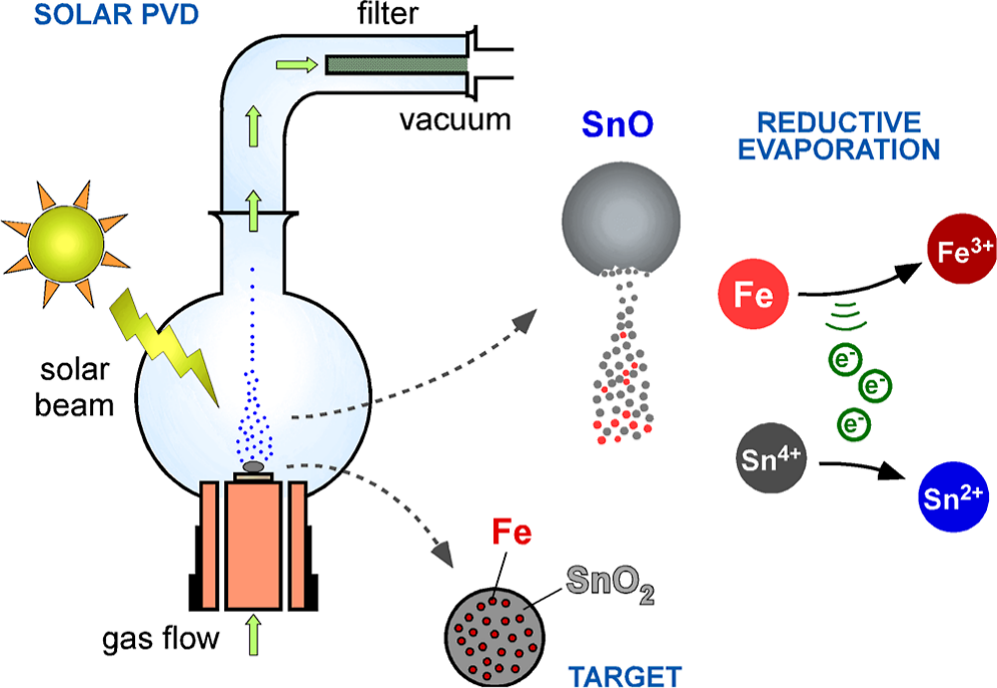
Schematic representation of the nanoparticle preparation setup and growth mechanism.

Nanopowders were prepared using the SPVD method in a Heliotron Pyrex glass chamber operated at the CNRS-PROMES 1.5 kW facility in Odeillo, France.^23^ The targets for each experiment were pressurized pellets of fine powdered SnO_2_ homogeneously mixed with varying percentages of pure Fe. More specifically, four samples were obtained by evaporation of pure SnO_2_ and SnO_2_/Fe at 25, 50, and 75 % wt Fe. Targets were placed in the focal zone of a 2 m diameter parabolic mirror with a power flux exceeding 10,000 suns. Evaporation process parameters were set to produce nanoparticles with primary particle sizes in the range of 20–80 nm at a vacuum base pressure of 5 × 10^–2^ mbar adjusted to 10 mbar by the continuous flow of Ar gas (99.999% purity, <2 ppm of O_2_). Nanoparticles formed during the rapid cooling of the fume were collected on a nanoporous alumina filter with the aid of a vacuum pump (Figure 1).

### 2.2 Nanopowder Characterization

X-ray diffraction (XRD) was used to identify the crystal structures and phase composition of the produced nanopowders. Measurements were performed using a Rigaku Ultima+ powder diffractometer with Cu Kα radiation, adjusted to a step size of 0.05°, a step time of 2 s, a voltage of 40 kV, and a current of 30 mA. Obtained diffractograms were analyzed by matching with the powder diffraction files (ICDD-PDF) database^24^ and quantified following the Rietveld refinement method using the Fullprof software.^25^ Transmission electron microscopy (TEM) images were recorded using a JEM-1210 (JEOL) microscope, operating at 120 kV, to determine the nanoscale morphology. Samples for TEM analysis were prepared by depositing a drop of the particle dispersion in isopropanol onto a carbon-coated copper grid. The hydrodynamic size distribution of the nanoparticles was obtained by a Horiba nanoPartica SZ-100V2 dynamic light scattering (DLS) analyzer, and the data were fitted by a log–normal distribution function. Fourier-transformed infrared (FT-IR) spectra of adsorbents were recorded in KBr media using a PerkinElmer Spectrum 100 spectrophotometer. For these measurements, the fine powder of the samples was dried and pelletized with excess KBr powder. The surface area of the samples was estimated by nitrogen gas adsorption at liquid nitrogen temperature (77 K) using a micropore surface area analyzer according to the Brunauer–Emmett–Teller (BET) model.

The macroscopic elemental analysis of the nanopowders was determined by graphite furnace atomic absorption spectrophotometry using a PerkinElmer AAnalyst 800 instrument following dissolution of a weighed sample in HCl. The reducing potential, as defined by the percentage of Sn^2+^, was determined by acid digestion of the solid followed by titration using KMnO_4_ solution. A 50 mg sample was dissolved under heat in 20 mL 7 M H_2_SO_4_ and then titrated with 0.05 M KMnO_4_. The end point of the titration was defined by the presence of a persistent weak pink color, indicating that the MnO_4_^–^ ions were no longer being reduced.

### 2.3 Cr(VI) Uptake Evaluation

The efficiency of developed nanopowders to capture Cr(VI) was evaluated by plotting the adsorption isotherms for the low concentration range, which covers the upcoming regulation limit of 25 μg/L. Batch adsorption experiments were performed by equilibrating a weighed quantity of nanoparticles with a Cr(VI) test solution. In addition, to simulate the performance in natural water, a challenge water spiked with common ions appearing in typical groundwater was used as a matrix, according to the composition suggested by the National Sanitation Foundation (NSF) standard.^26^ The natural-like water was prepared by the dissolution of the following reagents in 10 L of distilled water: 2.52 g (30 mmol) NaHCO_3_, 0.1214 g (1.4 mmol) NaNO_3_, 0.0018 g (0.0013 mmol) NaH_2_PO_4_·H_2_O, 0.0221 g (0.53 mmol) NaF, 0.706 mg (3.7 mmol) NaSiO_3_·5H_2_O, 1.47 g (10 mmol) CaCl_2_·2H_2_O, and 1.283 g (5.2 mmol) MgSO_4_·7H_2_O. By following this option, it is possible to overcome the wide variability of physicochemical parameters from site to site in natural water samples and provide universal data for a challenge water containing common interfering anions and cations at concentrations close to the average natural values. The composition of the test water is summarized in Table S3 in comparison to the corresponding parameters for the tap water of Thessaloniki. A typical experiment involved the dispersion of 20 mg of nanoparticles into 200 mL of aqueous Cr(VI) solution in 300 mL conical flasks. The dispersions were shaken for 24 h at 20 °C on an orbital shaker, and then the solid was separated by filtration using 0.22 μm pore-size membranes. Residual Cr(VI) concentration was determined by the diphenylcarbazide colorimetric method using a UV–visible spectrophotometer (Hitachi U-5100) adjusted at a wavelength of 540 nm.

To investigate the role of water pH in the adsorption efficiency, its value was fixed in a specific level in the range 6–8, where most natural waters are found, in a series of experimental sets. The pH was properly regulated by the addition of drops of 0.01 M HCl or NaOH solutions. Initial Cr(VI) concentrations were set below 1 mg/L, representing a range close to the regulation limits of drinking water. The adsorption isotherms were fitted by the Freundlich-type function, which describes better the observed trend in the low concentration range
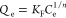
1where *Q*_e_ is the amount of Cr(VI) captured per mass of adsorbent, *C*_e_ is the equilibrium concentration, and *K*_F_ and *n* constants are related to adsorption capacity and affinity, respectively. The uptake capacity at a residual Cr(VI) concentration equal to the suggested drinking water regulation limit of 25 μg/L (*Q*_25_-index) was applied as a criterion for the evaluation of the performance of each tested sample. Fitting with a Langmuir function, which is characterized by a plateau formation when adsorption sites are saturated, was also performed as follows
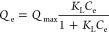
2where *Q*_max_ is the maximum monolayer adsorption capacity, and *K*_L_ is the Langmuir constant.

Separate experiments were carried out to study adsorption kinetics at 20 °C. A quantity of 20 mg nanoparticles was added to 200 mL of 1 mg/L Cr(VI) solutions adjusted to pH 7 and shaken for time intervals in the range of 0.1–48 h. Thermodynamic studies of the adsorption process were implemented by analyzing data from adsorption tests at pH 7 obtained at 5 and 35 °C by placing the flasks in a thermostatic cabinet and shaking for 24 h.

X-ray photoelectron spectroscopy (XPS) was used to confirm the Cr(VI) capture mechanism and the oxidation state of Sn in the nanoparticles. Particularly, spectra were acquired in a KRATOS Axis Ultra DLD system with a monochromated Al Kα X-ray beam employed as the excitation source. The pass energy was 160 eV for survey scans and 40 eV for high resolution spectra. Charging of specimens was avoided via a low-energy electron neutralizer, while calibration was performed by using the C 1s peak of adventitious carbon at 284.6 eV as the reference.

## 3 Results and Discussion

### 3.1 Adsorbents Characterization

**Figure 2:**
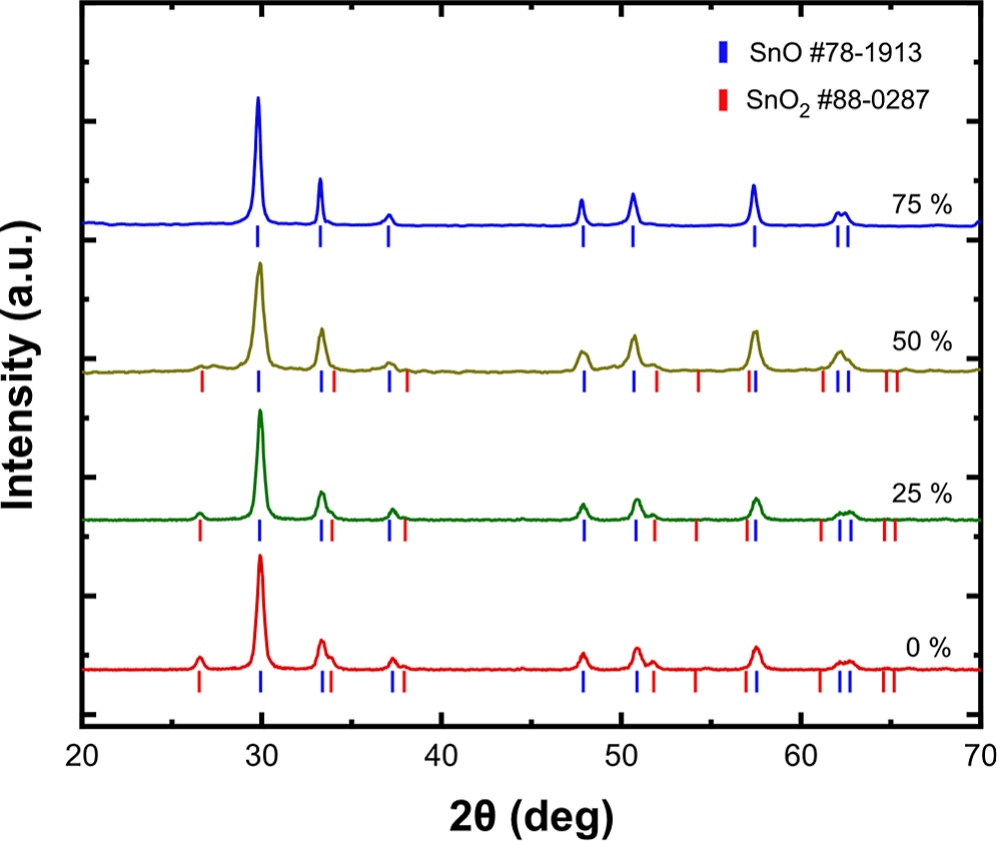
XRD diagrams of nanoparticles obtained by evaporating pure stannic oxide and SnO_2_ mixed with 25, 50, and 75% wt Fe.

Evaporation of pure SnO_2_ under the above-described conditions resulted in the production of nanopowders consisting mainly of SnO, as indicated by the corresponding XRD measurement (Figure 2). This is explained by the disassociation of the SnO_2_ during exposure to the concentrated beam and the condensation into a SnO structure due to the partial release of oxygen in the vapor phase. Such a process is also concomitant with the reduction of Sn^4+^ to Sn^2+^, although XRD also provides evidence for the presence of SnO_2_ in some of the samples, possibly located on the surface of the nanostructures. Quantification based on Rietveld analysis using database cards PDF#78-1913 (SnO) and PDF#88-0287 (SnO_2_) shows that the percentage of SnO_2_ reaches 30 % wt in the sample obtained from pure stannic oxide.

Considering the reduction of Cr(VI) as the favorable mechanism for its removal from water, diminishing the SnO_2_ coating layer would allow the maximum utilization of SnO potential. For this purpose, the addition of Fe to the target proved to be a sophisticated way of promoting the reduction of Sn^4+^ and inhibiting any SnO_2_ reformation (Figure 1). Indeed, the addition of at least 50% wt Fe was found to be adequate for the preparation of SnO nanoparticles free from the passive layer due to the electron donation role of Fe that occurs during target evaporation. At the same time, only trace amounts of Fe were detected in the prepared nanopowders after the total chemical analysis with atomic absorption (0.25, 0.32, and 0.80% wt, respectively, for the samples with 25, 50, and 75% wt Fe in the target), which suggests very different evaporation rates between Sn and Fe during the reduction process, in agreement with the findings of relevant studies.^27^ It should be noted that carrying out energy dispersive spectroscopy analysis combined with electron microscopy was not sufficient to localize such a low iron presence at the nanoscale. Similarly, the mentioned concentrations were below the XPS detection limit to estimate the oxidation state of iron. According to that, the Sn–Fe phase diagram demonstrates that the main phase above 910 °C is a Sn-rich liquid, which favorably facilitates its volatilization to SnO. Further evidence of the described growth mechanism was obtained by the XRD measurement of the residual target at the end of evaporation (Figure S1). The domination of the FeO phase (wustite), which is an intermediate oxidation product of iron, suggests the parallel existence of partial oxidation of the Fe powder during the evaporation of the tin phase.

**Figure 3:**
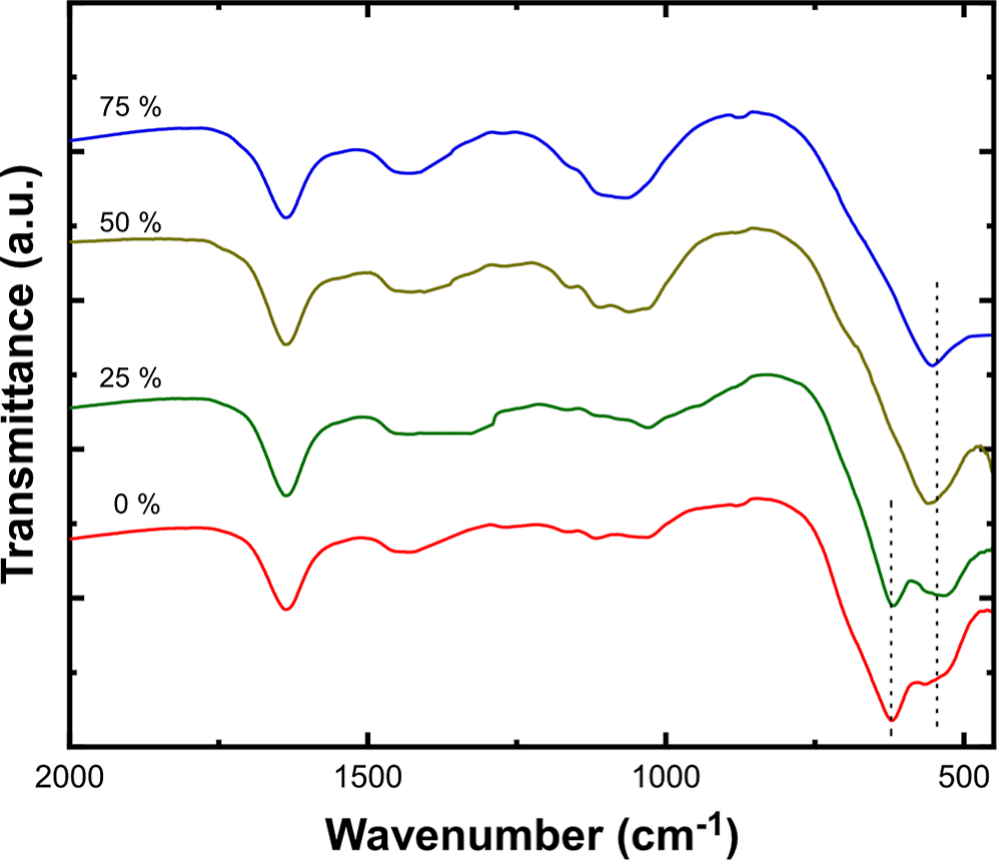
FTIR spectra of nanoparticles obtained by evaporating pure SnO_2_ and SnO_2_ mixed with 25, 50, and 75% wt Fe.

The evolution of nanoparticle composition, starting from the sole tin oxide evaporation to the increasing percentages of reducing iron additive, was also studied by determining the oxidation state of tin ions. Manganometry titrations indicated that the percentage of Sn^2+^ that implies the presence of SnO fluctuates above 90% of the total tin content for the nanoparticles prepared from 50 and 75% SnO_2_/Fe targets (93 and 95%, respectively), dropping to around 84% for the sample obtained by evaporation of the 25% SnO_2_/Fe target and 65% when pure SnO_2_ was used. These values are in agreement with the observed reducing role of iron during co-evaporation with SnO_2_. Additional confirmation was supplied from the FTIR spectra of samples (Figure 3). The nanoparticles collected by the evaporation of pure SnO_2_ and a mixture of low-content Fe are characterized by a double band at low wavenumbers with peaks at 620 and 530 cm^–1^. According to the literature, the first peak is assigned to the bulk Sn–O classical vibration A_1g_ mode of the SnO_2_ cassiterite phase, while the second peak can be attributed to surface Sn–O–Sn vibrations in the presence of oxygen vacancies and disordered nonstoichiometric romarchite (SnO).^28^ Such identification is consistent with the weakening and disappearance of the first peak, which correlates with the gradual decrease of SnO_2_ in reduced samples. Another peak observed for all samples at 1635 cm^–1^ corresponds to the bending vibration of H–O–H bonds as a result of water adsorption on the nanoparticle surface.^29^

**Figure 4:**
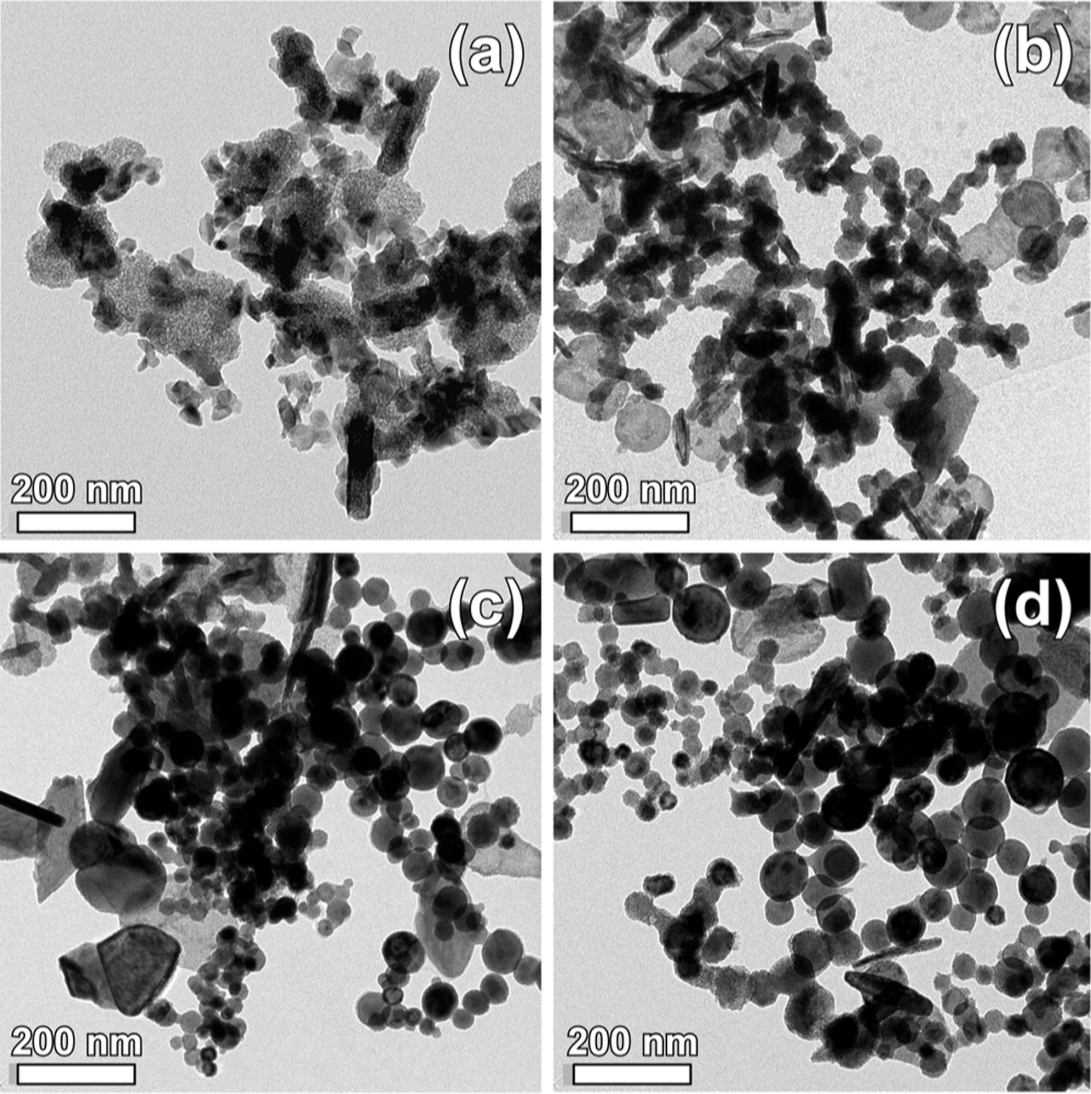
TEM images of nanoparticles obtained by evaporating pure SnO_2_ (a) and SnO_2_ mixed with 25 (b), 50 (c), and 75% wt Fe (d).

**Figure 5:**
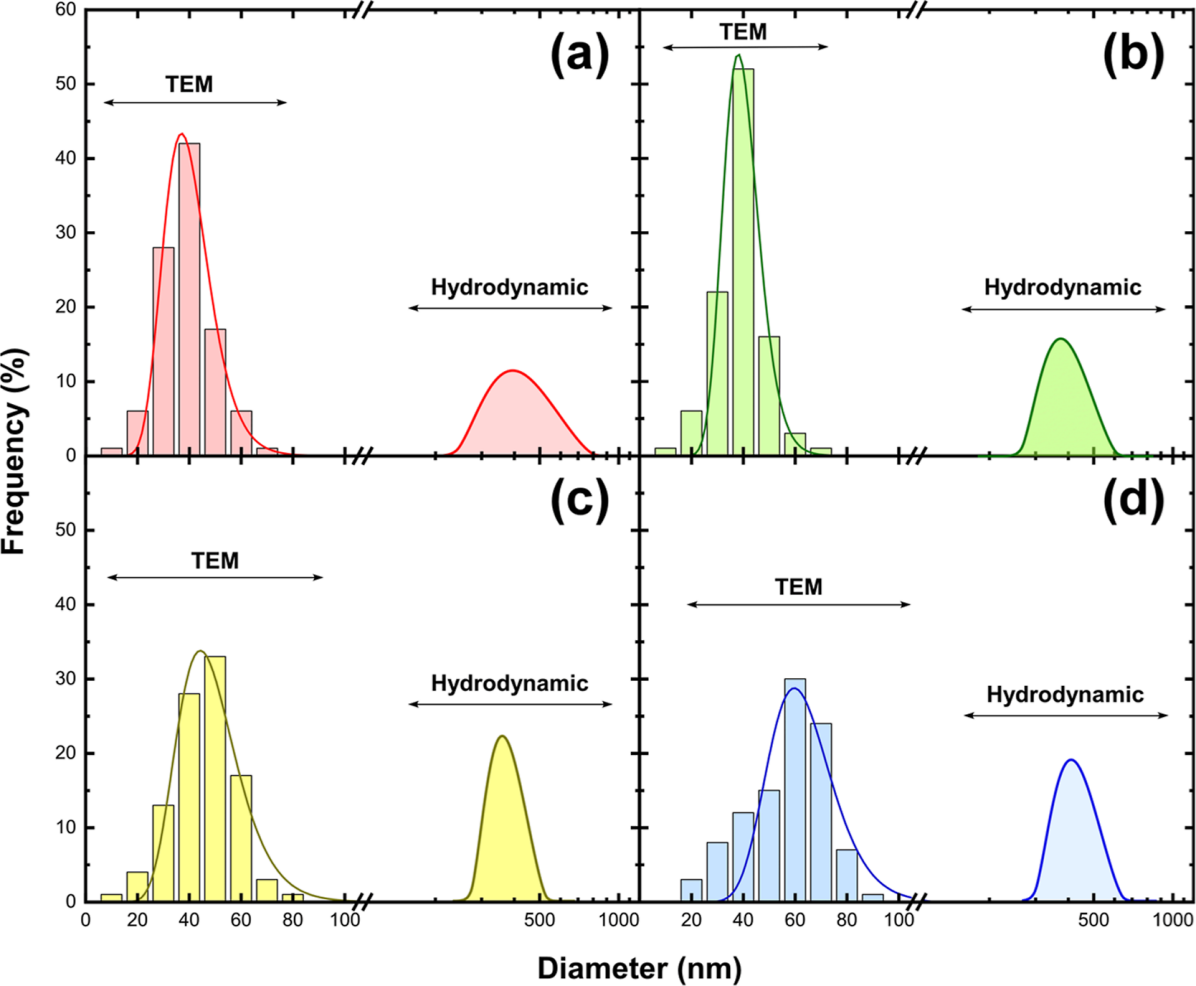
Size distributions of nanoparticles obtained by evaporating pure SnO_2_ (a) and SnO_2_ mixed with 25 (b), 50 (c), and 75% wt Fe (d), estimated by TEM images and DLS analysis; point to the logarithmic scale of the axis for hydrodynamic diameter.

Therefore, the selective growth mechanism of these particles is related to the evaporation process and the Fe content in the precursor target. In fact, electron microscopy observations (Figures 4 and 5) illustrate that evaporation of pure SnO_2_ pellets results mostly in polyhedroid nanocrystals with a size around 40 nm and larger acicular structures (Figure 4a), which is well consistent with the theoretical prediction that (110) planes have the lowest surface energy and may become a preferential growth direction.^30^ The addition of Fe appears to be responsible for the decrease of inhomogeneity of shape and the increase in average size. Correspondingly, the average nanoparticle diameter is around 40 nm when 25% wt Fe was used (Figure 4b) and approaches 50 nm for 50% wt Fe in the target (Figure 4c). After that, the volume increases gradually to form stable spherical particles,^31^ in our case, with an average diameter around 60 nm when excess Fe (75% wt) is introduced in the evaporating material (Figure 4d).

Corresponding specific surface area values were estimated to be 21 m^2^/g for pure SnO_2_ evaporates, reaching a maximum of 28 m^2^/g when 25% wt Fe was added, and then gradually dropping to 24 and 19 m^2^/g for the samples prepared with 50 and 75% wt Fe, respectively.

This finding matches with the XRD results and the fact that the SnO_2_ crystallizes into polymorphs of cassiterite, the rutile-like type phase (of space group *P*4_2_/*mnm*), while the most stable form of SnO is the so-called litharge tetragonal crystal structure (*P*4/*nmm*).^32^ In addition, it was previously found that, in general, tin oxides deposited at elevated partial pressures of oxygen consist of the rutile-like SnO_2_ form, while low pressures of oxygen result in SnO.^33^ Such morphological transformation may be attributed to oxygen vacancies due to partial deficiency of oxygen atoms, with reduction of some Sn^4+^ ions to Sn^2+^ as a possible charge compensation mechanism.^34^ Similar phenomena were also observed in the case of spherical and rod-shaped SnO_2_ particles^35^ for which a lower amount of oxygen vacancies was found in the nanorods; such particle types showed a higher (110) peak intensity.

The log-normal size distribution of nanoparticles obtained from electron microscopy images is presented in Figure 5. Hydrodynamic diameters estimated by DLS for powders dispersed in water are also included for comparison. For all samples, the average hydrodynamic size was found to be between 300 and 400 nm, suggesting a tendency for nanoparticle aggregation. However, those produced with pure SnO_2_ showed dispersion over a wider size range, whereas samples obtained with 50 and 75% SnO_2_/Fe targets appeared to have a lower standard deviation and aggregation extent despite their slightly larger mean diameter.

### 3.2 Cr(VI) Uptake

**Figure 6:**
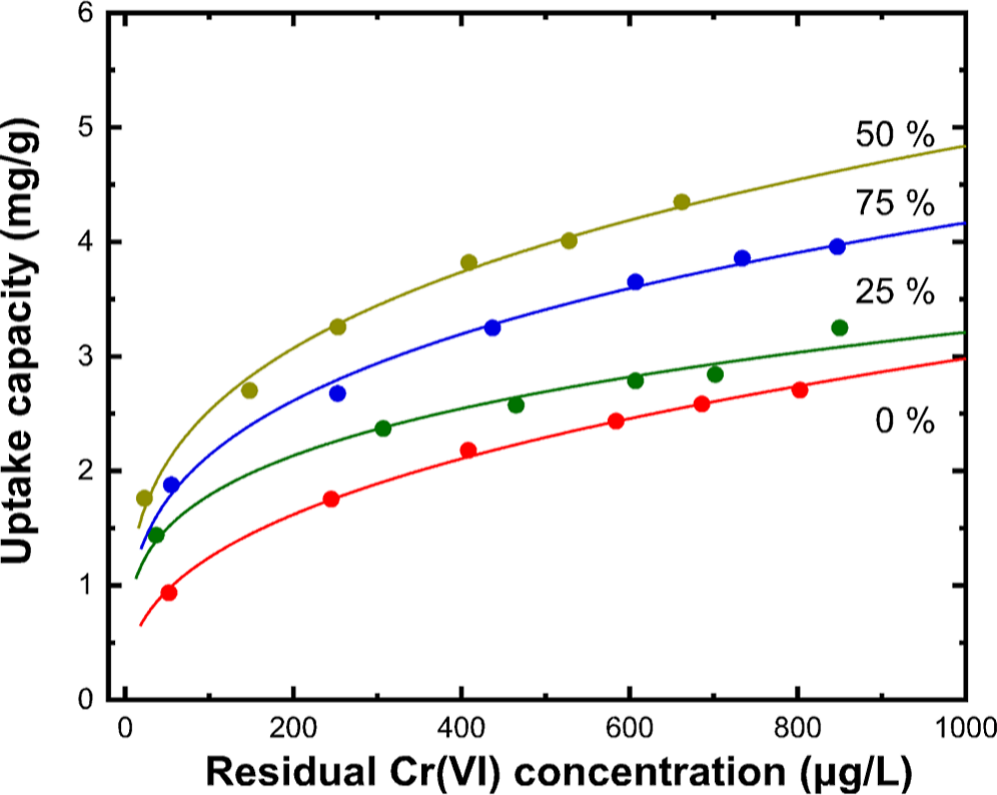
Cr(VI) removal isotherms in natural-like water at pH 7 and 20 °C for nanoparticles obtained by evaporating pure SnO_2_ and mixtures containing 25, 50, and 75% wt Fe. Continuous lines represent the Freundlich equation fitting of data.

Collected results from batch adsorption tests were used to plot the corresponding Cr(VI) removal isotherms for the studied nanoparticles (Figure 6). The sample prepared by evaporating SnO_2_/Fe 50% wt showed the highest performance to capture Cr(VI) from natural-like water at pH 7. The efficiency of this sample is even higher than that with 75 %wt Fe in the target; this suggests that above a percentage, any gain is balanced by the tendency to produce larger nanoparticles that provide less specific surface area and available adsorption sites. On the other hand, nanoparticles produced in the absence or with low Fe addition during evaporation offer a lower Cr(VI) capture capacity due to the significant presence of SnO_2_ in their composition, which inhibits the reduction mechanism.

Experimental data were fitted by Freundlich’s function to estimate the removal capacity of the nanoparticles at a residual concentration equal to the planned drinking water regulation limit of 25 μg/L (Q_25_ index). The solid lines in Figure 6 illustrate the fit results to the Freundlich model, whereas the corresponding parameters for the samples collected from pure SnO_2_ and SnO_2_ mixed with 50% wt Fe are included in Table 1. The value of the Q_25_ index for the sample prepared with 50% wt Fe in the target was found to be 1.74 mg/g, while those obtained with 75 and 25% wt Fe are 1.41 and 1.25 mg/g, respectively. In the absence of the reducing effect of Fe during nanoparticle condensation, the Q_25_ efficiency is reduced by one-third (0.57 mg/g). Fitting using Langmuir’s model appeared less accurate, indicating that Cr(VI) capture by the SnO nanoparticles is better described by a multilayer coverage of an heterogeneous particle surface with unlimited adsorption sites of unequal energies. Suggestively, the *Q*_max_ value from the Langmuir equation for the sample with 50% wt Fe in the target was estimated to be around 4.35 mg/g, whereas the corresponding value for pure SnO_2_ is around 3 mg/g (Table 1).

**Table 1:** Parameters of Freundlich and Langmuir Fitting on the Isotherms of Nanoparticles Obtained by Evaporating Pure SnO_2_ and Mixed SnO_2_/Fe

temperature *K*	Freundlich			Langmuir		
	*K*_F_ (μg_Cr_/mg)/(μg/L)^1/*n*^	1/*n*	*R*^2^	*K*_L_L/μg_Cr_	*Q*_ma*x*_μg_Cr_/mg	*R*^2^
Pure SnO_2_						
278	0.023	0.601	0.982	3.9 × 10^–3^	1.34	0.996
293	0.217	0.379	0.997	6.7 × 10^–3^	2.97	0.961
308	0.253	0.427	0.991	5.2 × 10^–3^	4.98	0.933
SnO_2_/Fe 50% wt						
278	0.036	0.530	0.993	4.3 × 10^–3^	1.36	0.993
293	0.323	0.379	0.997	7.3 × 10^–3^	4.35	0.953
308	0.373	0.391	0.997	7.7 × 10^–3^	5.24	0.932

### 3.3 Uptake Kinetic

**Figure 7:**
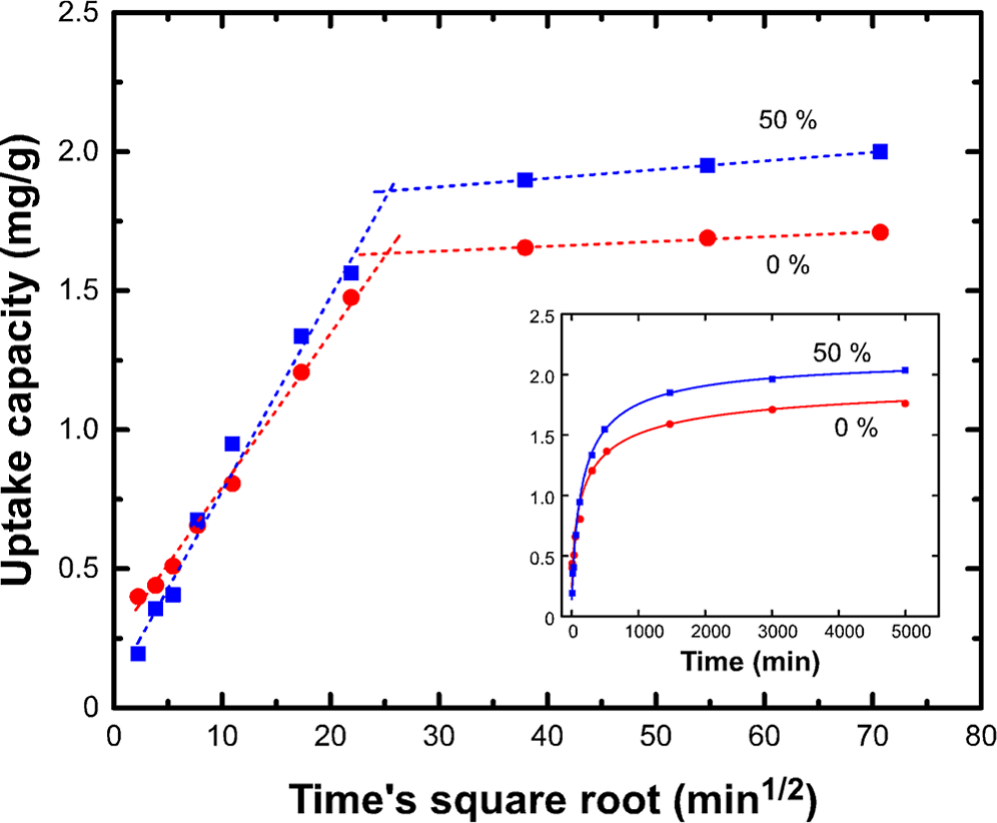
*t*^1/2^-dependence of Cr(VI) uptake capacity, at pH 7 and 20 °C, for nanoparticles obtained by evaporating pure SnO_2_ and its mixture with 50% wt Fe. Dotted lines indicate the linear fitting at intraparticle diffusion and equilibrium state regions. The inset shows the raw kinetic data.

Kinetic data were obtained for nanoparticles developed from pure SnO_2_ and SnO_2_ mixed with 50% wt Fe (Figure 7). Cr(VI) removal proceeded at a moderate rate during the first 6 h, with almost 60% captured, and then slowed further, reaching equilibrium after almost 40 h. This was best fitted by the pseudo-second order-model
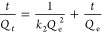
3where *k*_2_ is the rate constant for pseudo-second order rate kinetics (mg/μg**·**min), and *Q*_t_ and *Q*_e_ are the amount of Cr(VI) captured (μg Cr/mg adsorbent) at time t and at equilibrium, respectively. By plotting *t*/*Q*_*t*_ versus time, the values of *Q*_e_ and *k*_2_ were calculated from the slope and intercept, respectively. The value *k*_2_*Q*_e_^2^, which represents the initial sorption rate, was also calculated (Table 2). The pseudo-second order function has been widely applied to describe the removal of pollutants from water, considering a chemisorption step that involves both covalent forces and ion exchange.^36^

**Table 2:** Pseudo-Second Order and Intraparticle Diffusion Constant for Nanoparticles Obtained by Evaporating Pure SnO_2_ and SnO_2_ Mixed with 50% wt Fe

sample	*Q*_e_ μg/mg	*k*_2_*Q*_e_^2^ μg/mg·min	*k*_2_ mg/μg·min	*R*_2_^2^	*k*_*i*_ μg/mg·min^1/2^	*R*_i_^2^
pure SnO_2_	1.73	0.0215	0.00717	0.9930	0.056	0.9948
SnO_2_/Fe 50% wt	2.01	0.0186	0.00459	0.9944	0.071	0.9873

Since the suspension was vigorously agitated during experiments, it can be safely assumed that the rate-limiting step is intraparticle diffusion rather than mass transfer from the bulk liquid to the outer surface of the particle. To verify this assumption, Cr(VI) uptake data were plotted according to the parabolic diffusion law^37^
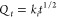
4where *k*_*i*_ is the diffusion rate constant (μg/mg/min^1/2^). The initial linear portion in the plot of Cr(VI) uptake versus *t*^1/2^ in Figure 7 corresponds to the intraparticle diffusion process, and the plateau is associated with the equilibrium state. Cr(VI) is initially adsorbed on the exterior surface of the adsorbent until saturation, while it later begins to gradually enter through the pores and interact with the interior surface of the nanoparticle volume. At the same time, intraparticle diffusion becomes the limiting rate step. The fact that intraparticle diffusion curves do not cross the origin of axes, along with the high positive value of the intercept of its linear regression equation, is indicative of the rapid adsorption of Cr(VI) onto the exterior surface of the adsorbent.

### 3.4 Thermodynamics of Uptake

To characterize the competition between these two (bulk and surface) processes, the effect of temperature on the Cr(VI) removal efficiency was analyzed, and the type of uptake process was interpreted based on thermodynamic parameters such as the Gibbs free energy (Δ*G*°), the enthalpy (Δ*H*°), and the entropy (Δ*S*°). The Gibbs free energy change of the adsorption reaction, Δ*G*° (J/mol), was estimated by
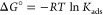
5where *R* is the ideal gas constant of 8.314 J/mol K, *T* is the absolute temperature in *K*, and *K*_ads_ the equilibrium adsorption constant, which can be approximated by Langmuir’s equilibrium constant *K*_L_ expressed in L/mol (Table 2).^38^ Δ*H*° and Δ*S*° were calculated from the van’t Hoff equation
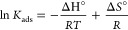
6by plotting ln(*K*_ads_) versus 1/*T* and estimating Δ*H*° and Δ*S*° from the slope and intercept, respectively. Tables 2 and 3 clearly show that when temperature is increased, the amount of Cr(VI) captured and the isotherm constants increased. The positive enthalpy change for both nanoparticles obtained by pure SnO_2_ and mixed SnO_2_/Fe targets indicates the endothermic character of the effect. More specifically, the enthalpy Δ*H*° value for nanoparticles obtained from pure SnO_2_ is significantly lower than that of SnO_2_/Fe, which implies a higher tendency of Cr(VI) oxy-anions to interact with the nanomaterial, providing higher reducing potential.

**Table 3:** Thermodynamic Parameters of Cr(VI) Uptake by Nanoparticles Obtained by Evaporating Pure SnO_2_ without the Addition of Fe

samples	Δ*Η*°kJ/mol	Δ*S*° J/mol·K	–Δ*G*°, kJ/mol		
			278 K	293 K	308 K
pure SnO_2_	9.35	78.5	12.3	14.2	14.6
SnO_2_/Fe 50% wt	15.1	99.9	12.5	14.8	15.5

An increase in randomness at the adsorbent/solution interface during adsorption is verified by the positive Δ*S*° for nanoparticles produced by SnO_2_/Fe evaporation, while, on the contrary, the entropy has a lower change for nanoparticles produced from pure SnO_2_. Furthermore, the negative values of Δ*G*° in the studied temperature range indicate the spontaneous nature of the process for both samples.

### 3.5 Effect of pH

**Figure 8:**
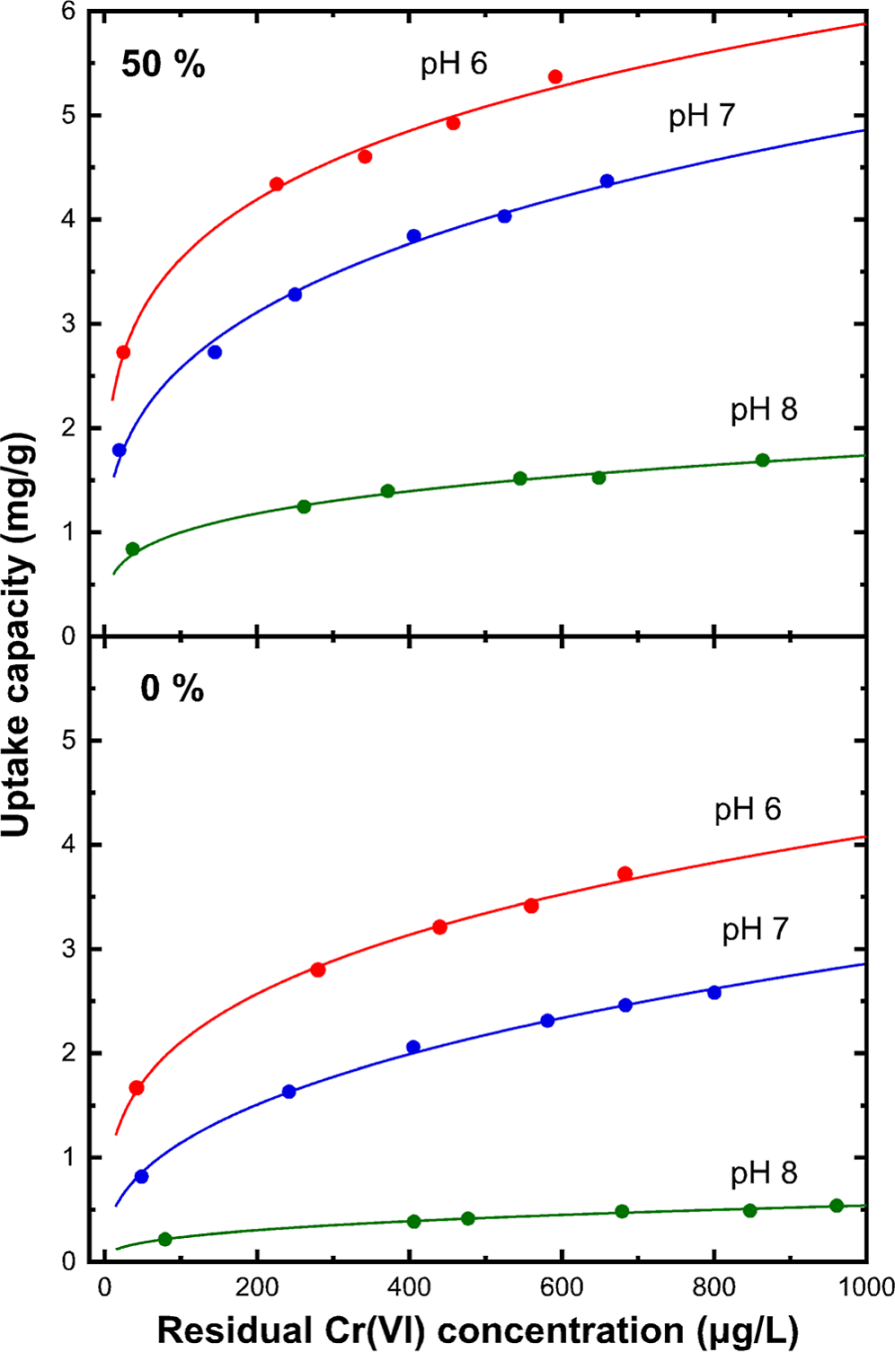
Dependence of Cr(VI) removal isotherms in natural-like water with equilibrium pH for nanoparticles obtained by evaporating pure SnO_2_ and after addition of 50% wt Fe. Continuous lines represent the Freundlich equation fitting of data.

In regard to the effect of pH, the acidity of treated water is commonly a critical parameter determining the efficiency of adsorption on solids since the correlation of surface charge with the speciation state of the targeted oxy-ion may vary. For metal oxide adsorbents, the increase of water acidity is responsible for the activation of more reduction sites on the surface due to the increase of the solubility of metal ions and their instant release from the outer shell. In addition to this, the isoelectric point of metal oxides and their hydrated forms, including the tin oxides, is usually observed between pH 6.5–7.5; this suggests that under acidic conditions, the surface of the adsorbent is positive. Figure 8 depicts the Cr(VI) uptake isotherms at different pH values. It is clearly seen that the reduction of Cr(VI) increased extremely with decreasing the solution pH. The sample containing SnO as the dominant phase showed an increase of the Q_25_ value by 55% when tested at pH 6, reaching 2.7 mg/g (Table S1). The corresponding value for the sample evaporated from pure SnO_2_ was 1.43 mg/g, doubling the respective value under pH 7. Such an observation is also attributed to the prominent presence of Cr(VI) in the form of HCrO_4_^–^ below pH 7, which requires one active surface site. Operating at pH 8 inhibits both the approach of Cr(VI) oxy-ions and the reduction to Cr(III), as denoted by the very low Q_25_ values of 0.71 and 0.15 mg/g. This is explained by the presence of abundant CrO_4_^2–^ species in combination with the negatively charged nanoparticle surface above the isoelectric point.

### 3.6 Capture Mechanism

XPS analysis of the sample prepared after evaporating the SnO_2_/Fe 50% wt target was carried out to define the oxidation state of Sn in the obtained nanoparticles, whereas the reduction mechanism during Cr(VI) was validated. Fitting of Sn 3d peaks for as obtained nanoparticles (Figure S5) using Gauss-Lorenz functions suggests the presence of a strong Sn^2+^ contribution identified at 486.3 and 494.8 eV for the 3d_5/2_ and 3d_3/2_ orbits, respectively, while a weak contribution (∼2%) attributed to elemental Sn^0^ is also signified at 484.9 and 493.7 eV. This observation is consistent with the reductive evaporation mechanism of nanoparticle growth in the catalytic presence of Fe in the target. The Sn^2+^ peak is clearly related to the dominant SnO structure of this sample. Quantification of XPS analysis, considering the major contributions by Sn 3d, C 1s, and O 1s (Table S2), may also validate the composition of the nanoparticles. Particularly, by assuming at least an oxygen molecular ratio of 1:1 with the surface carbon phases, the Sn-to-O mass ratio is estimated to be around 6.9, a value close to the stoichiometry ratio for the SnO (7.4). For comparison, the corresponding value for the Sn^4+^ oxide SnO_2_ would be 3.7.

**Figure 9:**
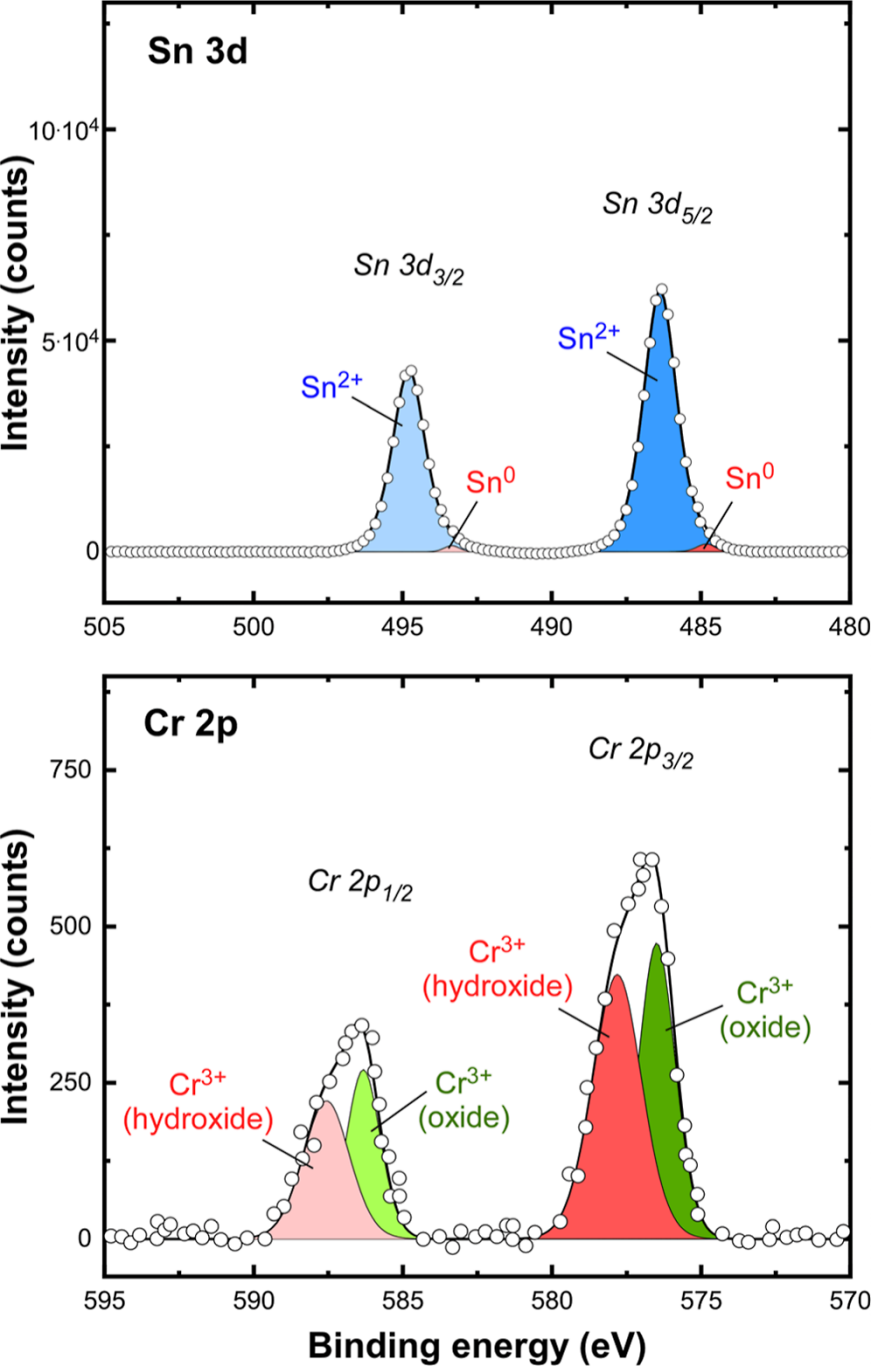
High-resolution XPS spectra of Sn 3d and Cr 2p orbits for nanoparticles obtained by evaporating SnO_2_ with 50% wt Fe after their use to purify water polluted with Cr(VI).

The pattern of the Sn 3d peaks is almost identical when the same sample is measured after being subjected to Cr(VI) remediation (Figure 9). The fitting peak positions were identified at the same binding energies for both SnO and Sn^0^ contributions, with only a small drop in the elemental phase percentage to around 1.5%. It seems that Cr(VI) uptake in low concentrations involves only a small percentage of sites, and therefore, it is not sufficient to provide any significant modification or severe surface oxidation of the nanoparticles surface. In this case, the Sn-to-O mass ratio was calculated to be around 6.7, slightly lower than the as-obtained sample.

Interpretation of the Cr 2p peaks provides more evidence on the mechanism of Cr(VI) capture by the SnO nanoparticles (Figure 9). More specifically, the 2p_3/2_ peak is deconvoluted into two contributions centered at 576.5 and 577.8 eV, both attributed to the existence of trivalent Cr, whether as oxide (Cr_2_O_3_-type) and hydroxide (Cr(OH)_3_-type) forms.^39,40^ Accordingly, during treatment of the polluted water, Cr(VI) oxyanions are completely reduced by the SnO nanoparticles to transform into Cr(III) precipitates. Depending on the proximity of Cr(III) to the surface, a significant part (65% wt) is dehydroxylated and enters the crystal structure of the tin phase, with the rest remaining loosely attached on the surface as hydroxide.^21^

## 4 Conclusions

Overcoming the surface passivation of tin oxide nanoparticles is a critical step toward their exploitation as competitive Cr(VI) adsorbents in drinking water facilities. In this work, the production of high-purity SnO nanoparticles was realized by a solar-assisted condensation method based on the co-evaporation of the tin source in the presence of iron powder. The catalytic role of iron lies in its reducing activity during the initial moments of evaporation and the much lower evaporation rate of iron, which inhibits its presence in the product. The SnO nanoparticles obtained in such a sophisticated way indicate a significant improvement of their Cr(VI) capture efficiency due to their high potential to decrease its toxicity, mobility, and oxidation state, turning Cr(VI) oxy-anions into Cr(III) insoluble oxides. Working with an iron content of 50% wt in the evaporating target resulted in pure spherical SnO nanoparticles, which showed the highest Cr(VI) removal capacity of 1.74 mg/g referring to residual Cr(VI) concentration below the upcoming regulation limit for drinking water.

## Supporting Information Available

Additional experimental results on the structure of the residual target, particle oxidation state, and temperature dependence of adsorption and fitting parameters (PDF)

## Notes

Based on experiments carried out at the CNRS-PROMES laboratory, UPR 8521, belonging to the French National Centre for Scientific Research (CNRS). Dataset related to this study acquired during access in NFFA facilities is available in http://doi.org/10.5281/zenodo.7589706.
