# Supplementary material for: Tin Oxide Nanoparticles via Solar Vapor Deposition for Hexavalent Chromium Remediation
Source: ACS Appl Nano Mater. 2023 Jul 7;6(15):13902–11. doi: 10.1021/acsanm.3c01567 (PMC10502794; doi:10.1021/acsanm.3c01567)
Supplement: Supplementary file 1 — an3c01567_si_001.pdf [file an3c01567_si_001.pdf]

Supporting information

Tin Oxide Nanoparticles via Solar Vapor Deposition for Hexavalent Chromium Remediation

Konstantinos Simeonidis[*1], Kyriaki Kalaitzidou[1], Theopoula Asimakidou[1], Carlos Martinez-Boubeta[2], Antonios Makridis[3], Anita Haeussler[4], Georgios Vourlias[3], Lluis Balcells[5]*

[1]Department of Chemical Engineering, Aristotle University of Thessaloniki, 54124 Thessaloniki, Greece

[2]Nanotech Solutions S.L., Ctra. Madrid 23, 40150 Villacastin, Spain

[3]Department of Physics, Aristotle University of Thessaloniki, 54124 Thessaloniki, Greece

[4]Processes, Materials and Solar Energy Laboratory, CNRS-PROMES, 7 Rue du Four Solaire, 66120 Font-Romeu, France

[5]Institut de Ciencia de Materials de Barcelona, CSIC, Campus Universitat Autònoma de Barcelona, A08193 Bellaterra, Spain

Corresponding author

*K.S., email: ksime@physics.auth.gr

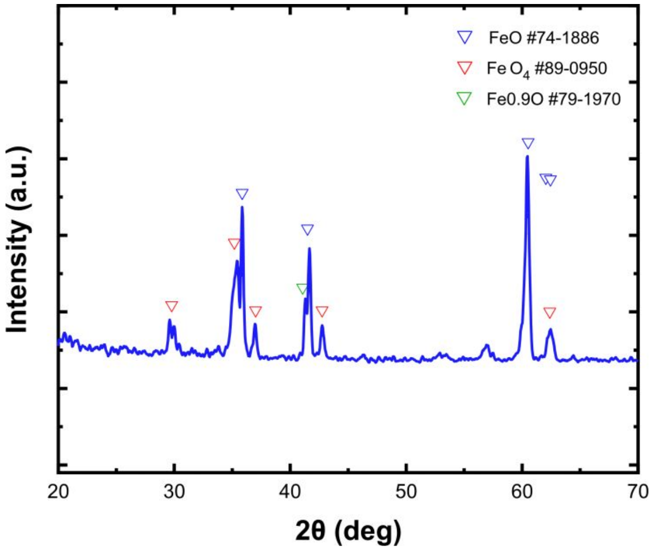

Figure S1. XRD diagram of the residual in the evaporation target obtained for SnO_2 mixed 50 %wt. Fe.

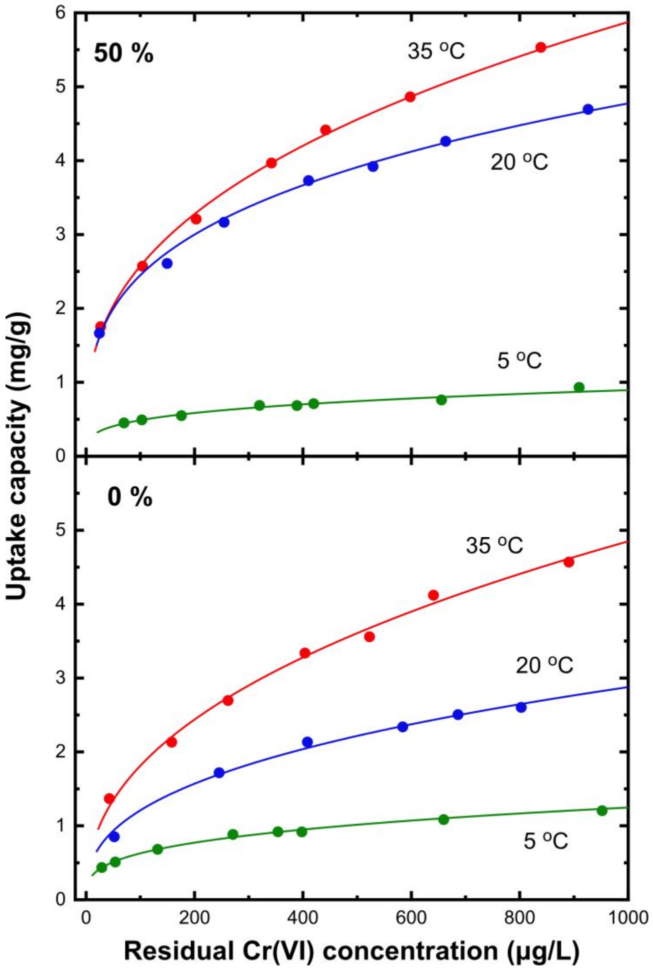

Figure S2. Temperature-dependence of Cr(VI) removal isotherms in natural-like water for nanoparticles obtained by evaporating pure SnO_2 and SnO_2 mixed with 50 %wt. Fe. Continuous lines represent the Freundlich equation fitting of data.

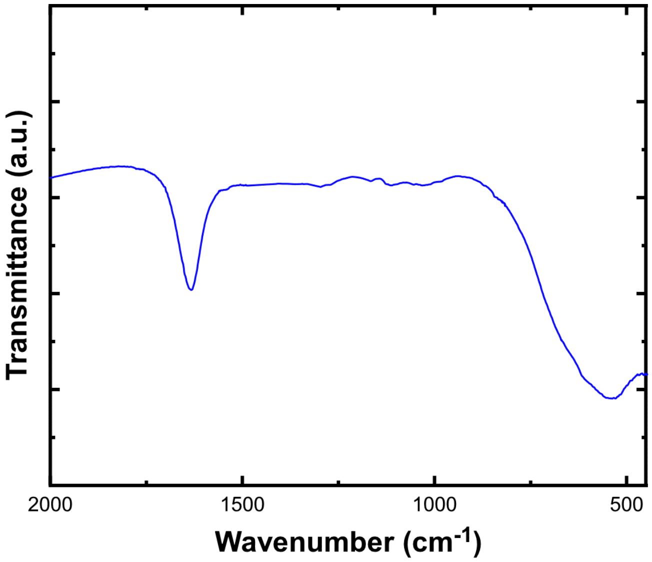

Figure S3. FTIR spectra of Cr(VI)-loaded nanoparticles obtained by evaporating pure stannic oxide and SnO_2 mixed with 50 %wt. Fe.

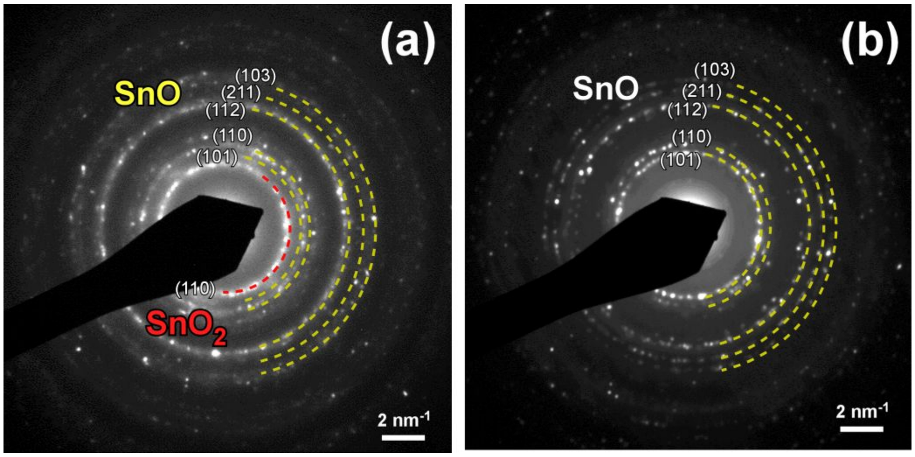

Figure S4. Electron diffraction patterns of nanoparticles obtained by evaporating pure SnO_2 (a) and SnO_2 mixed with 50 %wt. Fe (b).

Table S1. Parameters of Freundlich and Langmuir fitting on the isotherms at various equilibrium pH for nanoparticles obtained by evaporating pure SnO_2 and mixed SnO_2/Fe.

pH	Freundlich			Langmuir		
	K_F $(\mu g_{Cr}/mg)/(\mu g/L)^{1/n}$	1/n	R^2	K_L $L/\mu g_{Cr}$	Q_{max} $\mu g_{Cr}/mg$	R^2
**Pure SnO_2**						
6	0.561	0.287	0.997	$16.7 \cdot 10^{-3}$	3.75	0.928
7	0.217	0.379	0.997	$6.7 \cdot 10^{-3}$	2.97	0.961
8	0.045	0.358	0.959	$2.9 \cdot 10^{-3}$	0.72	0.963
**SnO_2/Fe 50 %wt.**						
6	1.376	0.210	0.994	$41.9 \cdot 10^{-3}$	5.14	0.918
7	0.323	0.379	0.997	$7.3 \cdot 10^{-3}$	4.35	0.953
8	0.329	0.240	0.997	$6.8 \cdot 10^{-3}$	1.93	0.957

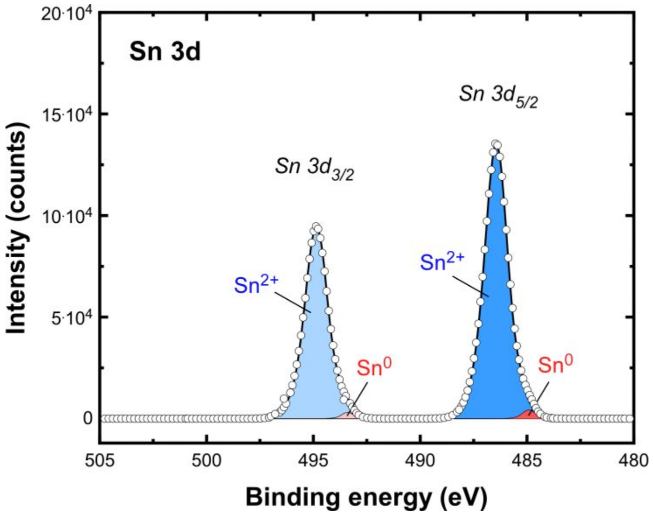

Figure S5. Sn3d high resolution XPS spectrum of as obtained nanoparticles prepared by evaporating SnO_2 mixed with 50 %wt. Fe.

Table S2. Quantification of XPS measurements for nanoparticles prepared by evaporating SnO_2 mixed with 50 %wt. Fe, before and after purification of water polluted with Cr(VI).

Element	As obtained %wt.	After Cr(VI) uptake %wt.
C	5.57	9.85
O	18.53	22.92
Sn	75.90	65.59
Cr	-	1.64

Table S3. Composition of the NSF challenge water versus tap water in Thessaloniki.

Cations	NSF mg/L	Tap mg/L	Anions	NSF mg/L	Tap mg/L
Na^+	88.8	78.1	HCO_3^-	183	325.4
Ca^{2+}	40	50.4	SO_4^{2-}	50	62.7
Mg^{2+}	12.7	28.3	Cl^-	71	62.6
			NO_3^-	2	-
			F^-	1	-
			PO_4^{3-}	0.04	0.2
			SiO_2	20	15.9
